# Enhancing the Dyeability of Polypropylene Fibers by Melt Blending with Polyethylene Terephthalate

**DOI:** 10.1155/2013/468542

**Published:** 2013-10-30

**Authors:** Fereshteh Mirjalili, Siamak Moradian, Farhad Ameri

**Affiliations:** ^1^Department of Polymer Engineering and Color Technology, Amirkabir University of Technology, P.O. Box 15875-4413, Tehran, Iran; ^2^Centre of Excellence for Color Science and Technology, Institute for Color Science and Technology, P.O. Box 16765-654, Tehran, Iran; ^3^Department of Color Physics, Institute for Color Science and Technology, P.O. Box 16765-654, Iran

## Abstract

Attempts were made to modify polypropylene fibers by melt blending with polyethylene terephthalate in order to enhance the dyeability of the resultant fiber. Five blends of polypropylene/polyethylene terephthalate/compatibilizer were prepared and subsequently spun into fibers. Three disperse dyes were used to dye such modified fibers at boiling and 130°C. The dyeing performance of the blend fibers, as well as the morphological, chemical, thermal, and mechanical properties, of the corresponding blends was characterized by means of spectrophotometry, polarized optical microscopy, scanning electron microscopy (SEM), FT-IR spectroscopy, differential scanning calorimetry (DSC), and tensile testing.

## 1. Introduction

Polypropylene (PP) exemplifies a class of polymeric materials called polyolefins. This thermoplastic polymer has had a remarkable growth over the past 60 years. The reasons for this growth have been the versatility of the polymer; ability of the polymer to be modified and tailored for specific applications which arise from its overall balance of physical, mechanical, electrical, chemical, and thermal properties; and last but not least a competitive price. Additionally, polypropylene-based materials have very good stability over a wide temperature range. For these reasons, there seems to be a bright future for polypropylene as a building material in the present century. Composites based on this polymer are also of great utility because these types of materials combine inherent cost effectiveness of polypropylene with desirable attributes of a wide spectrum of commodity thermoplastics [1]. From the application viewpoint, a great volume of PP finds its way into an area that may be classified into fibers and fabrics, including carpet backing, upholstery fabrics, clothing, geotextiles, disposable diapers, medical fabric, and automotive interior fabrics. The advantages offered by PP fiber include low specific gravity, which means greater bulk per given weight, strength, and chemical and stain resistances [2]. However, as an olefinic textile fiber, polypropylene lacks polar groups and is regarded as a hydrophobic polymer with a moisture regain of less than 1%. These attributes as well as high crystallinity limit the accessibility of colorant molecules especially dyestuffs to the interior of the fiber [3]. Today, the most convenient and highly productive method for coloring polypropylene fibers is the mass pigmentation technique in which a masterbatch of pigment is added to polymer before processing. However, there are some disadvantages associated with this technique including lack of high chroma colors, inability to correct by redyeing, appearance of color streaks, and frequent yarn breaking during the finishing processes [3, 4]. Therefore, in order to modify polypropylene to make it dyeable from aqueous dyebaths, a considerable research in recent years had been undertaken. Improving the dyeability of polypropylene fibres by means of chemical and physical modification methods inclusive of surface chemical reactions and functionalization, copolymerization and graft polymerizations, plasma and gamma radiation treatments, and blending of PP with polar polymers, metal compounds, inorganic nanoparticles, particularly, nanoclays, dendrimers and hyperbranched polymers, and many other additives has been widely investigated [5–13]. Considering economic, feasibility, and environmental aspects, it seems that melt blending with different kinds of polymeric additives such as polystyrene, polyamides, and polyesters, prior to spinning, could be an efficient process for the dyeability modification of PP fibers [14–16]. Amongst these polymers, polyesters especially polyethylene terephthalate (PET) offer some advantages over the others including improvement in dyeability, as well as enhancing the mechanical properties of the resultant fiber [17–19]. On the other hand, currently there is considerable concern regarding the running out of raw materials and a renewed intent in conserving natural resources and increasing recycling. In addition, there is a growing interest in PET recycling due to the widespread use of this polymer in the packaging industries mainly as beverage bottles, jars, video and audio tapes, and food containers. PET does not create a direct hazard to environment, but due to its substantial fraction by volume in the waste stream and its high resistance to the atmospheric and biological agents, it is considered as a noxious material [20]. Therefore, imparting dyeability to PP fibers by collection, disintegration, granulation, and recirculation of waste polyethylene terephthalate can be a promising potential for recycling and reusing PET wastes in PP/PET blend fibers. However, PET and PP are incompatible due to their differences in polarity and chemical nature. Therefore, without compatibilization their blends exhibit a clear two-phase morphology, where the dispersed phase forms relatively large spherical droplets with no particular adhesion between the phases, and thus the desired mechanical properties cannot be achieved. Hence, appropriate compatibilizers are needed to attain better adhesion between the two phases [21, 22]. It has been shown by many researchers that compatibilization of PP/PET blends through the addition of thermoplastic maleic anhydride grafted copolymers such as acrylic acid (AA), maleic anhydride (MA), and glycidyl methacrylate (GMA) grafted polyolefins and modified SBS block copolymers provides the means to significantly enhance the properties of the melt blend during extrusion, modifies their interfacial properties, and stabilizes the morphology of resultant blends. However, the compatibilizers are low-molecular-weight compounds in comparison with blend partners and incorporation of uncontrolled quantities of these additives may degrade the mechanical strength of the blend [23–25].

In a previous work [26], we employed a mathematical and statistical technique to find an optimal region for the amount of each component of a tripartite PP/PET/maleic anhydride grafted polypropylene (PP-g-MA) blend, in such a way that the resulting blend showed acceptable dyeability toward disperse dyestuffs, as well as appropriate mechanical strength. As was concluded in our previous publication, the weight fractions of PET and PP-g-MA in the blend should be in the range of 10–15 wt% and 4-5 wt%, respectively, to guarantee a modified polypropylene with desired dyeability and mechanical properties. In addition, it was found that a PET : PP-g-MA ratio of about 3 : 1 can serve this purpose. In the present investigation, attempts were made to perform a more comprehensive study on the dyeing performance of some disperse dyestuffs toward PP/PET blend fibers with varying amounts of PET and PP-g-MA inside and outside of the previously obtained optimal range. For this purpose, morphological, chemical, thermal, and mechanical properties of the resulting PP/PET/PP-g-MA blends were characterized by means of scanning electron microscopy (SEM), FT-IR spectroscopy, differential scanning calorimetry (DSC), and tensile testing. Furthermore, the dyeing performance of the corresponding blend fibers was evaluated by means of spectrophotometry, polarized optical microscopy, and assessment of fastness properties of the dyed modified fibers.

## 2. Experimental

### 2.1. Materials

*Homopolymer Moplen V30S*, a fiber grade polypropylene (PP) produced by the Arak Petrochemical Co. (Arak, Iran) with melt flow rate (MFR) of 18 g/10 min (2.16 kg, 230°C), *TG-641*, a textile grade polyethylene terephthalate (PET) provided by Shahid Tondguyan Petrochemical Co. (Khuzestan, Iran) with intrinsic viscosity of 0.64 dL/g, and a DuPont commercial product *Fusabond P MD353D*, a maleic anhydride grafted polypropylene (PP-g-MA) with MFR of 450 g/10 min (2.16 kg, 190°C) as compatibilizer were used in all experiments. In order to prevent thermal degradation of PP during melt blending and melt spinning processes, a thermal stabilizer *Irganox 1010* was purchased from the Ciba-Geigy Chemical Materials Company (Basel, Switzerland). In addition, three disperse dyestuffs, namely, C. I. Disperse Blue 60, C. I. Disperse Yellow 211, and C. I. Disperse Red 234, were selected to investigate the dyeability of blend fibers in a wide range of color shades. 

### 2.2. Preparation of Specimens

The PP/PET/PP-g-MA blend compositions in this study were coded and formulated according to Table 1. 

The samples in Table 1 are coded according to their PET content. For instance, B12 refers to the blend containing 12 wt% PET. These selected blend compositions are based on the results obtained from an experimental design presented in our previous study [26], in which an optimum PET : PP-g-MA ratio of about 3 : 1 was found to provide a PP/PET/PP-g-MA blend with acceptable dyeability and mechanical properties.

Before blending, PET was dried at 120°C, and PP and the compatibilizer were dried at 80°C for 12 hours in a vacuum oven. Melt blending of dry-mixed materials was performed in a Brabender Plasti-Corder twin screw extruder under rotor speed of 30 rpm. The temperature settings of the barrel were 255, 260, 270, 270, and 260°C. The amount of thermal stabilizer used in each composition was 0.5 wt% based on the weight of polypropylene in the blend. All five blends were subsequently spun into fibers using a laboratory single screw extruder equipped with a spinneret having one 3 mm diameter orifice. The drawing of blend fibers was carried out at room temperature with a total draw ratio of 1400. 

### 2.3. Morphology Characterization

Morphology of the cryogenically fractured cross-sections of the blend fibers was characterized by means of a *Philips XL30* scanning electron microscope (SEM). 

### 2.4. FT-IR Spectroscopy

In order to demonstrate the compatibilizing performance of PP-g-MA in the PP/PET blend, FT-IR spectroscopy characterization was performed. The FT-IR spectra were recorded on a Bomem MB154S FT-IR Spectrometer (Quebec, Canada) from 3500 to 450 cm^−1^. For each sample, a 100 *μ*m thin film was prepared by compression moulding at 260°C.

### 2.5. Thermal Analysis

Differential Scanning Calorimetry (DSC) under a nitrogen atmosphere was carried out on a Shimadzu DSC-60 calorimeter (Kyoto, Japan). A scanning rate of 10°C min^−1^ was used, and the sample weight was 5-6 mg. The samples were heated to 300°C and then cooled. The transition temperatures were taken as the maximum peaks in the calorimetric curves. The percent degree of crystallinity (*X*_*c*_) corresponding to the PP component in each blend was calculated from the ratio Δ*H*_*m*_/Δ*H*_*m*_^0^, where Δ*H*_*m*_ and Δ*H*_*m*_^0^ are the apparent and the fully crystalline heats of fusion, respectively. For pure polypropylene, Δ*H*_*m*_^0^ is 209 J/g.

### 2.6. Dyeing Procedure

The characteristics of three blue, yellow, and red disperse dyes used for dyeing of the blend fibers are shown in Table 2.

Apart from representing primary colors in order to render an extensive color gamut, these three disperse dyes were selected in such a way that their levels of activation energy were equal. All three disperse dyes have medium activation energies. Therefore, acceptable dyeability of the resulting blend fibers toward these dyes assures a higher dyeability of blend fibers with disperse dyes of lower activation energies. On the other hand, it is clear that dyeing of blend fibers with dyes of higher activation energies would result in lower dye uptake. 

**Figure 1:**
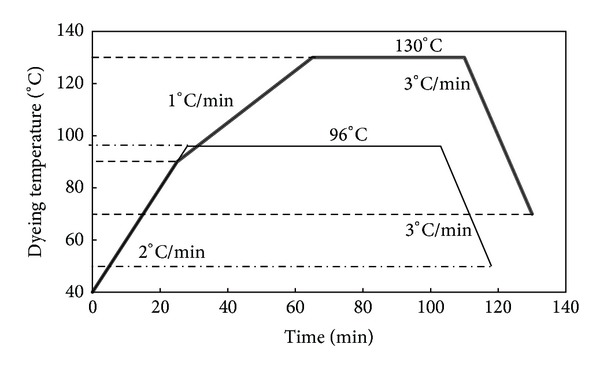
Dyeing profiles at boiling (*≈*100°C) and 130°C.

However, the effect of the molecular size and chemical structure of dye molecules on their dyeing ability should also be considered. All blend fibers were pretreated with 5 g/L nonionic detergent (Lotensol from Hansa Co., Stuttgart, Germany) at 80°C for 20 min using a liquor-to-goods ratio of 50 : 1, after which the fibers were rinsed and dried. Dyeings were carried out in a Rota-Dyer dyeing machine (supplied by the Nasaj Sanat Yazd Co., Yazd, Iran) at 1% omf as the saturation concentration of dyes on blend fibers which was already determined, according to dyeing profiles shown in Figure 1. The dyeing pH was adjusted to 4-5 with acetic acid. Moreover, in order to prevent uneven dyeings, a liquor-to-goods ratio of 100 : 1 was employed for all dyeings. 

As depicted in Figure 1, dyeings were carried out at two temperatures, namely, boiling (96°C) and 130°C, in order to inspect the effect of temperature on dyeing performance of the blend fibers. At the end of each dyeing, excess dye on the surface of the fiber was removed by subjecting the dyed fibers to a reduction clearing process. Reduction clearing was performed at a liquor-to-goods ratio of 50 : 1 using 2 g/L sodium hydrosulphite, 1 g/L sodium hydroxide, and 1 g/L of a nonionic detergent for 20 min at 50°C. The reduction cleared samples were then rinsed and allowed to dry. For comparison purposes, pure PP and pure PET fibers were also separately pretreated and dyed under the same conditions described for the blend fibers.

### 2.7. Measurement of Dye Uptake

In order to determine the dye uptakes of dyed fibers, the single constant Kubelka-Munk coefficients (*K*/*S*) were determined. For this purpose, spectral reflectance factors in the range of 360 to 750 nm were obtained using a GretagMacbeth ColorEye 7000A spectrophotometer with 8°/diffuse illumination and observation geometry, respectively, together with the UV and the specular components included mode for the measurements. Thereafter, the K/S values corresponding to the wavelength of maximum absorption (*λ*_max⁡_) for each dyestuff were reported as a dye uptake parameter. In addition, CIELAB colorimetric coordinates were calculated by the instrument software using CIE standard illuminant D65 and the CIE 1964 standard colorimetric observer.

### 2.8. Polarized Optical Microscopy Characterization

The distribution of dye molecules within the blend fibers and the effect of the dyeing temperature on blend fiber's dye uptakes were investigated visually by means of a polarized optical microscope. Polarized optical microscopy experiments were conducted on a Zeiss KF2 microscope connected to a digital image recorder. The fibers were parallelly mounted in Canada balsam mounting resin and then cut into very thin layers by means of a surgery blade. 

### 2.9. Color Fastness Properties of Dyeing

The light fastness of all dyeings was determined according to ISO 105-B02:1994(E), using a Xenotest 150S (Heraeus Hanau) light fastness apparatus. In addition, the wash fastness of the dyeing was also measured according to ISO 105-C02:1989(E).

### 2.10. Measurement of Mechanical Properties

In order to determine the tensile properties of the blends and the effect of incorporation of different amounts of PET and the compatibilizer on the mechanical properties of the resultant PP fibers, a tensile test was performed according to the ASTM D882 standard on 300 micron films of the blends at a crosshead speed of 5 mm min^−1^ by means of a Galdabini 1890 tensile testing machine (Cardano al Campo, Italy). Three 10 × 50 × 0.3 mm^3^ films were utilized in each tensile test provided using a compression moulding process at 260°C, and the average values of the triplicate samples were reported.

## 3. Results and Discussion

### 3.1. Morphology

#### 3.1.1. Morphology of the Blends

**Figure 2:**
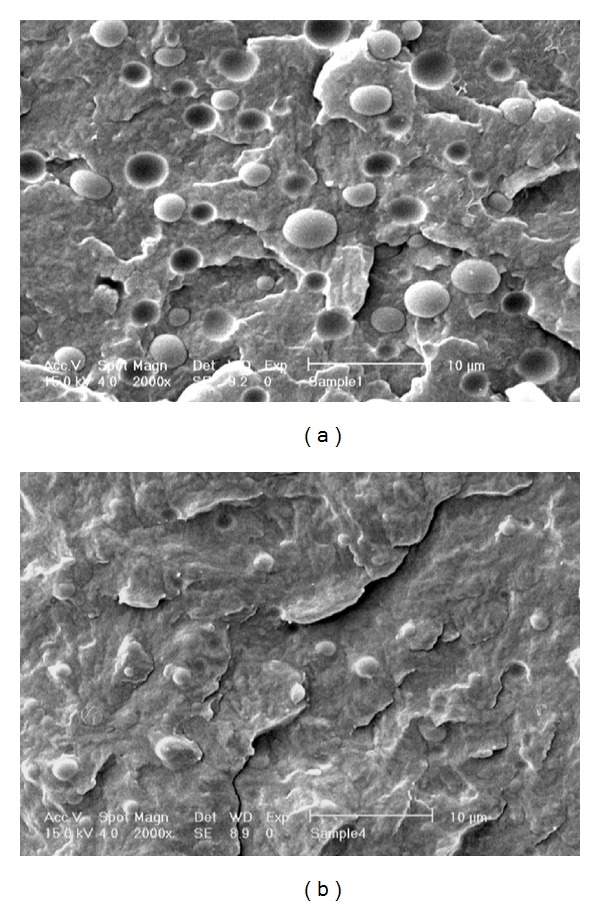
SEM micrographs of uncompatibilized and compatibilized blends (magnification: ×2000). (a) PP/PET/PP-g-MA: 80/20/0; (b) PP/PET/PP-g-MA: 73/20/7.

In order to evaluate the performance of PP-g-MA as a compatibilizer in the PP/PET blends, SEM micrographs of compatibilized B20 blend (PP/PET/PP-g-MA: 73/20/7) and its equivalent uncompatibilized blend (PP/PET: 80/20) are shown in Figure 2.

It can be seen that the uncompatibilized blend shows an obvious two-phase morphology, in which the average diameter of dispersed PET particles is about 4 microns. But by adding the compatibilizer to the blend, the average diameter of the dispersed phase has decreased. This reduction in particle size could have arisen from chemical reactions between maleic anhydride (MA) groups of PP-g-MA and functional terminal –OH (or –COOH) groups of PET which cause the generation of an in situ PP-g-MA-PET copolymer during the melt blending process. This graft copolymer accumulates at the blend interfaces, reduces the interfacial tension, and prevents the coalescence of dispersed particles. In addition, adhesion between the two incompatible phases and consequently the mechanical properties of the resultant blend improves as a result of compatibilization. Considering the dyeability, compatibilization also enhances the dye uptake by minimizing the size of PET particles, increasing the amount of interfaces, and reducing the degree of crystallinity of the blend and, therefore, increasing the number of accessible active sites for fixation of dyestuff molecules. 

#### 3.1.2. Morphology of the Blend Fibers

**Figure 3:**
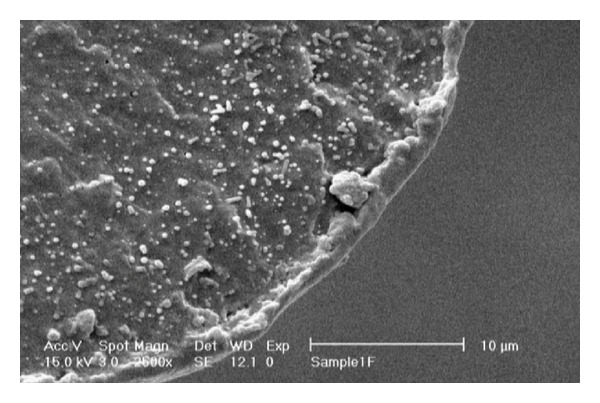
SEM micrograph of B12 blend fiber, magnification: ×2500.

Figure 3 shows the SEM micrograph of fractured cross-section of B12 blend fiber with 12 wt% PET. 

As can be seen in Figure 3, the produced blend fiber is based on PET microfibrils embedded in polypropylene matrix. This “fibril-matrix” morphology is a result of the rheological behavior of polypropylene and polyethylene terephthalate. The viscosity ratio of PET and PP is less than 1. For that reason, fine PET dispersed particles are deformed to microfibrils during the spinning and drawing processes. Therefore, a fibril-matrix morphology is obviously observed in the case of PP/PET blend fibers. These microfibers can enhance the mechanical strength of in situ formed fiber/matrix composite [18, 19]. The micrographs also illustrate that the average diameter of PET fibrils is about 100 nm that is much smaller than the size of particles in equivalent blends before spinning. Therefore, it is expected that a blend fiber would be capable of absorbing a larger number of dye molecules in comparison with an unspun one. Besides, the other important point to notice in SEM micrographs is that the fine microfibrils are distributed uniformly throughout the fiber, and this is crucial to obtain the dyeing uniformity (i.e., level dyeing). 

### 3.2. FT-IR Characterization

**Figure 4:**
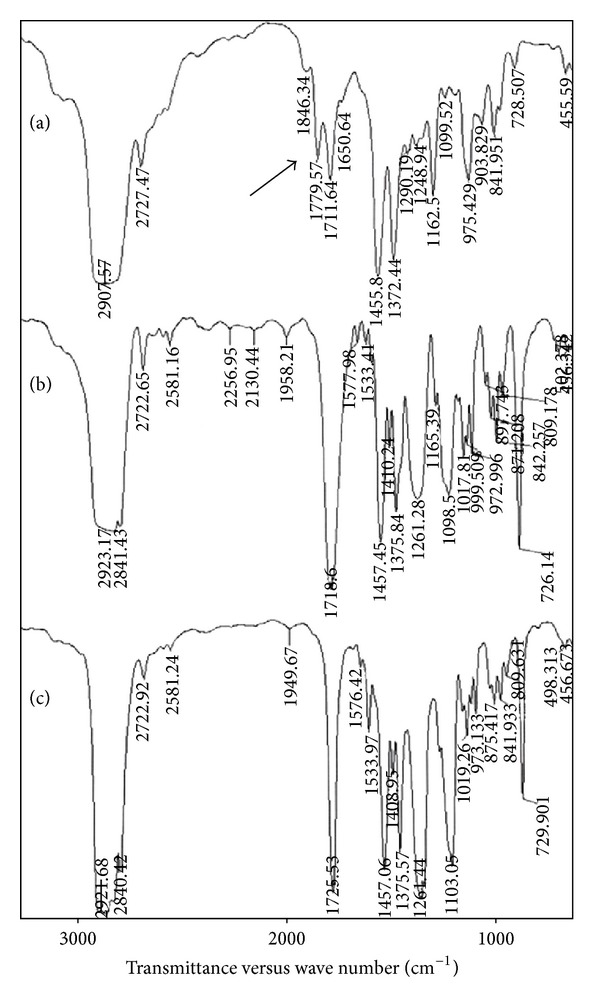
FTIR spectra of (a) PP-g-MA; (b) uncompatibilized; and (c) compatibilized blends.

It is accepted by many researchers that maleic anhydride (MA) functionalized compatibilizers would enhance the miscibility of the two incompatible polymers through in  situ chemical reactions between MA and functional end groups of polymers. In the case of PP/PET blends, –OH or –COOH functional groups of PET can participate in the compatibilization reactions with MA groups of PP-g-MA. To confirm these reactions, the FT-IR spectra of neat and blend polymers were investigated.  Figure 4 shows the FT-IR spectra of pure PP-g-MA, uncompatibilized 80/20 PP/PET, and compatibilized 73/20/7 PP/PET/PP-g-MA blends. 

The absorption bands of the carbonyl group for PP-g-MA were at 1711 and 1779 cm^−1^ (see Figure 4(a)). However, despite the presence of 7 wt% of PP-g-MA in the blend, both absorption bands disappeared in the spectrum of the PP/PET/PP-g-MA 73/20/7 blend which may uphold the hypothesis of chemical reactions between MA and –OH and –COOH terminal groups of PET. 

### 3.3. Thermal Analysis

The differential scanning calorimetry (DSC) results for neat PP and PP components in five blends are listed in Table 3.

The data in Table 3 show that the melting temperatures (*T*_*m*_) of PP in the blends are almost the same as that in the pure state. All the melting temperatures are about 168°C. This result is easy to grasp if it is remembered that PP is the major phase in these blends. However, the melting enthalpies (Δ*H*_*m*_) of PP component are obviously lower than that of the neat PP, implying lower degrees of crystallinity of the PP phase in the blends. The Δ*H*_*m*_ for the neat PP is 100.5 J/g, which means a 48.1% degree of crystallinity according to the melting enthalpy of 209 J/g for the fully crystalline PP.

As mentioned in the literature [19, 23, 27], in the uncompatibilized PP/PET blends, increasing the PET content would benefit the crystallization of PP due to the nucleating effect of the second polymer. Therefore, *T*_*c*_ of PP in the uncompatibilized blend is higher than that of neat PP. However, in the case of compatibilized blends, the crystallization of PP would be restricted as a result of the interactions between the blend components and the compatibilizer molecules. It is observed from *T*_*c*_ data in Table 3 that the crystallization temperatures (*T*_*c*_) of PP component increase with increasing PET content, which indicates that PET has a heterogeneous nucleation effect on PP crystallization. Therefore, it may be concluded that the spherulite size of PP has decreased and the number of spherulites has increased. The degree of crystallinity (*X*_*c*_) data and crystallization enthalpies (Δ*H*_*c*_) indicate that all blends are less crystalline than the virgin PP. This reduction in crystallinity of PP/PET/PP-g-MA blends can be ascribed to mobility restriction of PP chains due to in  situ formation of chemical bonds between PP and PET via PP-g-MA as revealed by FT-IR spectra. 

### 3.4. Dyeing Properties of the Blend Fibers

#### 3.4.1. Dye Uptake

**Figure 5:**
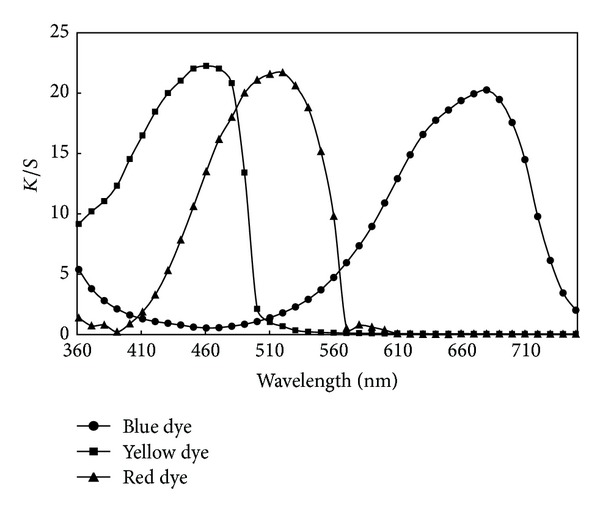
K/S curves of pure PET fiber dyed with three disperse dyestuffs at boiling (*≈*100°C).

For specifying the wavelength of maximum absorption (*λ*_max⁡_) for each disperse dye on pure and blend fibers, the variation of spectral reflectance factors and K/S values in the visible spectrum range from 360 to 750 nm was considered. The wavelength at which the spectral reflectance factor is minimal and K/S value is maximal represents *λ*_max⁡_. As an instance, the K/S curves of pure PET fiber, dyed with the three utilized blue, yellow, and red disperse dyes at boiling are shown in Figure 5. The K/S values are calculated using the single constant Kubelka-Munk equation [26].

As can be seen, the absorption maxima of blue, yellow, and red disperse dyes lay at 680, 460, and 520 nm, respectively. For almost all of the other dyeings, these three wavelengths also represent the wavelength of maximum absorption for the blue, yellow, and red disperse dyes. The K/S values at *λ*_max⁡_, calculated using the single constant Kubelka-Munk equation for all dyed fibers, are presented in Table 4. 

The K/S parameter, namely, the ratio of the absorption coefficient to the scattering coefficient, is indicative of the amount of dye molecules absorbed into the fiber. The higher the K/S value, the greater the dye uptake and therefore the deeper the color shades. The K/S data in Table 4 clearly show that for both dyeing temperatures, dye uptake of all blend fibers is much higher than that of pure polypropylene fiber. However, there is a small difference between the K/S values corresponding to each blend fiber and that of pure polyethylene terephthalate fiber. This increase in dye uptake of PP/PET blend fibers can be ascribed to the incorporation of ester functional groups and aromatic rings to polypropylene matrix through blending them with polyethylene terephthalate. These groups can function as active sites for absorbing and fixing dye molecules within the modified fiber. Besides, as the thermal analysis of the blends shows, the decrease in total crystallinity of the blend fiber and formation of additional amorphous regions facilitate the diffusion of dye molecules into the fibers. 

Moreover, it can be seen that the K/S values increase as the PET concentration in the modified PP fibers increases. However, the difference between the dye uptakes of five blend fibers containing varying amounts of PET additive is of lower significance. 

The other interesting result obtained from the K/S data is that modification of PP fiber by means of melt blending with PET has enhanced the affinity of all three disperse dyes with different chemical structures and color shades to modified fibers. In other words, it seems that this modification method can provide PP fibers that are dyeable in a wide range of color shades and consequently capable of producing an extensive color gamut. 

#### 3.4.2. Effect of Dyeing Temperature on Color Coordinates

The CIELAB colorimetric coordinates of dyed pure and blend fibers inclusive of lightness (L*), color-opponent coordinates a* and b*, chroma (C*), and hue (*h*) using D65 standard illuminant and 10° standard observer, for 100 and 130°C dyeing temperatures, are shown in Table 5.

As can be seen in Table 5, at each dyeing temperature, the lightness value of blend fibers decreases as the PET content increases. This dwindling trend in lightness values is indicative of increased color strength of the samples and is in good agreement with corresponding K/S data in Table 4.

**Figure 6:**
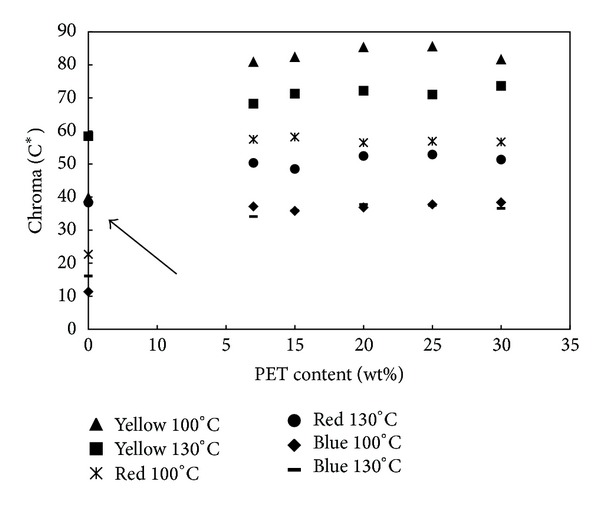
Variation of chroma (C*) with PET contents of dyed PP/PET blend fibers.

In addition, in spite of the negligible difference between a*, b* values and therefore hue of pure PET and blend fibers, the corresponding hues of pure PP fiber are somewhat different from the others. The duller color of pure PP fibers was completely obvious by visual comparison of the samples. However, the most important result derived from colorimetric data is that in comparison with blend fibers, pure PP fiber has the lowest chroma values for all three dyes. In other words, modification of polypropylene by addition of polyethylene terephthalate enhances the purity of the resultant color.  Figure 6 presents the variation of chroma, namely, the color purity of the dyed samples as a function of PET content for three disperse dyestuffs. It can be seen in Figure 6 that yellow and blue disperse dyes have the highest and the lowest chroma values on the blend fibers, respectively. 

It is generally known that the polymeric structure of any synthetic fiber has a large influence on the diffusion of dye molecules into the fiber. The “free volume model” [28] can provide a simplified version of actual conditions. This model is based on the concept of thermal movement of macromolecules within the fiber. Application of external energy causes the breaking of intermolecular connections, and hence the mobility of the molecular segments is increased. 

It has been shown that increasing the dyeing temperature above the glass transition temperature (*T*_*g*_) of polymeric fibers causes increased flexibility of the polymer segments and an increase in the amount of voids available for the dye diffusion process [29]. Therefore, it is expected that increasing the dyeing temperature would enhance the amount of absorbed dye molecules. It can be seen from Table 4 that the K/S values, taken to be an indication of the dye uptake, increase as temperature increases from 100°C to 130°C. The reduction in lightness values (L*) at the raised temperature confirms this result. 

In contrary to enhanced dye uptake, it can be seen from Figure 6 that the color purity of the five blend fibers almost declines with increased dyeing temperature where this decrease is most obvious for yellow dyed fibers. This could possibly be attributed to scattering of PET oligomers at the surface dulling the resultant color. 

However, what is actually important here is that almost all blend fibers dyed at boiling have chroma values comparable to or higher than those of pure PET fiber (see Figure 6), whereas the blend fibers dyed at 130°C have lower chroma values in comparison with pure PET. The color purity is often a desirable attribute from the customer's point of view. On the other hand, in order to conserve energy, dyeing at 100°C is definitely more preferable. Therefore, considering the small differences between the K/S values of blend fibers and that of pure polyethylene terephthalate fiber, it appears that in contrast with polyester fibers which should be dyed at high temperatures, namely, 130°C or higher to achieve acceptable dye uptakes and color saturations, modification with PET would yield PP/PET blend fibers that are satisfyingly dyeable at boiling temperature of water. 

#### 3.4.3. Fastness Properties

The fastness ratings for blend fibers dyed with three disperse dyes at 100°C are presented in Table 6. 

Table 6 indicates that the wash fastness ratings of all dyed blend fibers were very good to excellent. However, light fastness was good to very good (5-6). The good fastness properties of PP/PET blend fibers dyed with disperse dyestuffs can be attributed to good fixation of dye molecules into the fibrous material. In other words, blending of polypropylene with polyethylene terephthalate can efficiently provide permanent active sorption sites within the resultant fiber to immobilize dye molecules. 

### 3.5. Polarized Optical Microscopy

**Figure 7:**
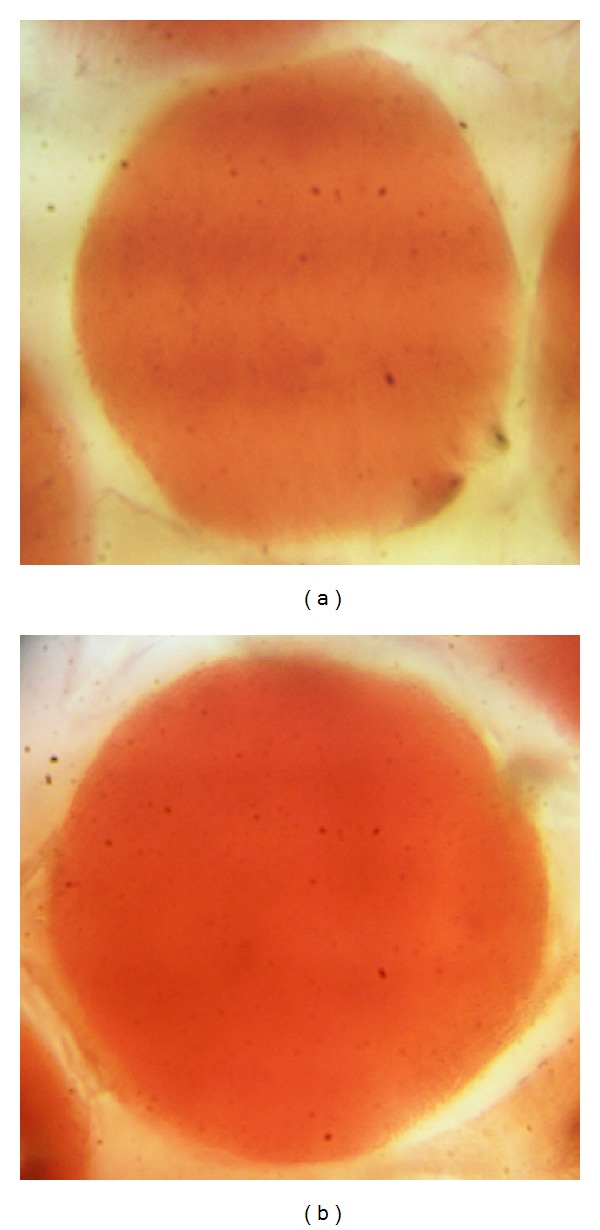
Polarized optical micro images of cross section of dyed B30 blend fiber at (a) 100°C; (b) 130°C.

The images captured from the cross-section of B30 blend fiber after dyeing with 1% omf red disperse dye at 100 and 130°C, using the polarized optical microscope are shown in Figure 7.

Figure 7 clearly shows that the B30 blend fiber dyed at 130°C has higher color strength in comparison with the fiber dyed at boiling which is in agreement with K/S data of Table 4. However, the most important result obtained from optical microimages is the completely uniform distribution of dye molecules in the fiber, indicating the efficiency of melt blending and compatibilization processes which lead to a good distribution of PET domains within PP matrix, as was previously discussed (see Figure 3). 

### 3.6. Mechanical Properties

It is generally agreed that polypropylene, as one of the most versatile engineering thermoplastic polymers, has moderate mechanical properties. Therefore, in order to extend the field of applications of this material, further deterioration in mechanical properties must be prevented. Polymer blending, on the other hand, offers a promising route to generate novel materials with better properties than the individual ones. In this field, the blends of polypropylene and polyethylene terephthalate are of interest since the latter can improve the stiffness and modulus of the relatively weak partner. However, like most polymer blends, a PP/PET blend is incompatible giving poor mechanical properties due to the weak interfacial adhesion between the two components. It has been shown by many researchers that the modification of interfacial regions by the addition of compatibilizers can enhance the mechanical properties of the resultant blend. The tensile strength and tensile modulus of neat PP, neat PET, and all five blends are given in Table 7. 

As can be seen in Table 7, the mechanical strength of PP has improved as a result of incorporation of PET in the presence of the compatibilizer. All blends have higher tensile modulus than neat PP due to the much higher stiffness and modulus of PET. It can be observed from Table 7 that the tensile modulus of pure PET is 2571.25 MPa which is considerably greater than that of polypropylene (1062.54 MPa). In addition, there is an increasing trend in tensile modulus of PP/PET blends with increasing the PET content. 

Investigating the tensile strength data in Table 7 shows that compatibilized blends possess higher strength than neat PP. It means that, addition of such an extremely strong polymer in the presence of a suitable compatibilizer can interestingly reinforce the weaker polypropylene. However, it should be noted that incorporation of high contents of low-molecular-weight PP-g-MA may worsen the mechanical strength of the blend. It can be seen that B12 and B15 PP/PET/PP-g-MA blends with respective compositions of 84/12/4 and 80/15/5 have higher tensile strength than pure PP. However, increasing the weight fractions of PET and PP-g-MA above these values has weakened the tensile strength of the blend, as B25 and B30 blends have lower tensile strength values in comparison with neat polypropylene. This behavior can be explained by the fact that high contents of low-molecular-weight PP-g-MA and incompatible PET as well as the weak interactions between PET dispersed particles and PP matrix, caused by inefficient compatibilization, result in deterioration of the strength of the blend. 

## 4. Conclusions

The K/S values of dyed PP/PET/PP-g-MA blend fibers with disperse dyestuffs clearly showed that dye uptake of all blend fibers is much higher than that of pure polypropylene fiber at both 100 and 130°C dyeing temperatures. Moreover, it was found that the K/S values increase as the PET content increases. The K/S values also interestingly show that the dyeability of all modified PP fibers is comparable to neat polyester fiber. Spectrophotometric investigation of dyed fibers revealed that modification of polypropylene by addition of polyethylene terephthalate enhances the purity of the resultant color. However, a slight decrease in color purity was observed with increasing the dyeing temperature from 100 to 130°C, enunciating that PP/PET blend fibers are satisfyingly dyeable at the boiling. Assessments of dyeing fastness properties of all dyed blend fibers demonstrated a very good to excellent wash fastness and a good to very good light fastness. SEM micrographs of fractured cross-section of the blends showed that all blends have a fine morphology in which PET is distributed in PP matrix in the form of spherical particles as the dispersed phase. Moreover, the characterization of the morphology of the blend fibers by means of SEM micrographs implicated a fibril-matrix morphology in the form of PET microfibrils distributed uniformly within the fiber. The efficiency of melt blending and compatibilization processes and then the appropriate distribution of PET domains in PP matrix were clearly obvious from polarized optical microimages of cross-sections of dyed blend fibers in which the dye molecules were distributed completely uniform within the fiber. The results of thermal analysis confirmed the lower degrees of crystallinity (*X*_*c*_) of the blends than neat PP. In addition, investigating the mechanical properties of blends showed that the tensile modulus of PP/PET blends increases with increasing the PET component's concentration. Tensile strength data showed that compatibilized blends possess higher strength than neat PP. However, it was found that increasing the weight fractions of PET and PP-g-MA above 20 and 7 wt%, respectively, weakens the tensile strength of the blend. On the basis of these results, we may be able to conclude that modification with polyethylene terephthalate via melt blending, in the presence of a compatibilizer, offers a promising procedure to overcome the problem of poor dyeability of polypropylene and to remarkably enhance the affinity of disperse dyestuffs toward this nonpolar olefinic fiber. 

## Figures and Tables

**Table 1:** Coding and formulations of five blend fibers containing various amounts of PET.

Sample	PET (wt%)	PP-g-MA (wt%)	PP (wt%)
B12	12	4	84
B15	15	5	80
B20	20	7	73
B25	25	8.5	66.5
B30	30	10	60

**Table 2:** Characteristics of disperse dyestuffs used in the experiment.

Commercial name	Generic name	Chemical structure	Activation energy level
Terasil Blue BGE	C. I. Disperse Blue 60	Anthraquinone	Medium
Terasil Yellow 4G	C. I. Disperse Yellow 211	Monoazo	Medium
Terasil Red R	C. I. Disperse Red 324	Monoazo	Medium

**Table 3:** DSC data for the PP component in the PP/PET blends.

Sample	*T*_*m*_ (°C)	Δ*H*_*m*_ (J/g)	*T*_*c*_ (°C)	Δ*H*_*c*_ (J/g)	*X*_*c*_ (%)
PP	168.5	100.5	111.2	118.6	48.1
B12	167.8	95.0	112.1	112.1	45.2
B15	167.5	92.5	114.2	108.7	41.0
B20	168.6	89.6	115.5	105.0	38.6
B25	167.5	88.3	114.8	103.3	39.4
B30	166.2	86.5	116.7	104.6	40.7

**Table 4:** The *K*/*S* values of dyed pure and blend fibers with three disperse dyes at boil and 130°C.

Sample	C. I. Disperse Blue 60		C. I. Disperse Yellow 211		C. I. Disperse Red 324	
	*K*/*S* (boil)	*K*/*S* (130°C)	*K*/*S* (boil)	*K*/*S* (130°C)	*K*/*S* (boil)	*K*/*S* (130°C)
PP	0.98	1.65	3.13	7.27	1.27	4.8
B12	16.54	19.49	19.45	22.74	19.18	21.58
B15	17.46	20.82	19.23	23.96	19.96	22.57
B20	18.37	21.56	20.92	23.64	19.87	22.60
B25	18.57	21.34	21.85	24.08	20.16	22.45
B30	19.08	22.96	21.64	24.53	20.50	22.65
PET	20.3	24.88	22.15	26.18	21.09	25.62

**Table 5:** Colorimetric attributes of dyed samples under D65 standard illuminant and 10° standard observer.

Sample		Blue disperse dye					Yellow disperse dye					Red disperse dye				
	Temp. (°C)	L*	a*	b*	C*	*h*°	L*	a*	b*	C*	*h*°	L*	a*	b*	C*	*h*°
PP	100	72.98	−4.47	−10.42	11.34	246.79	80.76	−0.96	39.78	39.8	91.39	68.78	19.67	11.26	22.67	29.79
	130	66.87	−3.03	−15.81	16.10	259.13	71.40	5.80	58.11	58.40	84.29	50.63	33.90	17.90	38.34	27.83
B12	100	50.11	−18.21	−32.35	37.12	240.61	78.25	11.84	79.95	80.82	81.57	39.39	52.85	22.50	57.45	23.06
	130	38.34	−11.16	−32.21	34.09	250.88	59.26	17.67	65.89	68.22	74.98	33.48	44.88	22.73	50.31	26.86
B15	100	47.86	−21.28	−28.80	35.81	233.53	73.94	12.65	81.49	82.33	81.03	37.92	52.57	24.81	58.13	25.26
	130	42.32	−9.69	−34.60	35.93	254.35	60.56	13.03	70.99	71.30	79.60	32.14	43.25	21.93	48.49	26.88
B20	100	47.78	−18.77	−31.71	36.85	239.38	71.83	14.33	84.07	85.29	80.32	39.34	50.99	24.22	56.45	25.41
	130	41.37	−8.90	−36.63	37.69	256.33	64.99	15.82	70.38	72.14	77.32	34.30	47.04	23.13	52.42	26.18
B25	100	45.48	−17.32	−33.55	37.76	242.70	72.91	10.73	84.86	85.53	82.79	36.13	51.34	24.48	56.88	25.49
	130	35.12	−7.88	−36.65	37.49	257.85	66.22	11.86	70.02	71.02	80.38	33.45	47.51	23.18	52.86	26.01
B30	100	44.88	−18.33	−33.72	38.38	241.47	71.57	13.77	80.40	81.57	80.27	38.71	51.04	25.15	56.67	26.56
	130	43.43	−9.46	−35.32	36.57	255.00	64.82	10.85	72.85	73.65	81.52	32.99	46.06	23.16	51.34	26.12
PET	100	42.9	−16.22	−35.16	38.72	245.22	68.97	15.08	80.47	84.60	80.62	37.54	52.41	25.38	58.24	25.83
	130	39.38	−13.74	−37.23	39.69	249.73	70.75	12.54	97.41	87.22	82.66	34.37	51.18	27.81	58.25	28.51

**Table 6:** Color fastness to washing (ISO 105-C02:1989(E)) and light (ISO 105-B02:1994(E)).

Sample		Wash fastness							Light fastness
		Color change	Staining					
			Acrylic	Nylon	Acetate	Cotton	Wool	Polyester
B12	Blue	4-5	5	5	5	5	5	5	6
	Yellow	5	5	5	5	5	5	5	6
	Red	4-5	5	5	5	5	5	5	5

B15	Blue	4-5	5	5	5	5	5	5	5-6
	Yellow	4-5	5	5	5	5	5	5	6
	Red	5	5	5	5	5	5	5	5

B20	Blue	4-5	5	5	5	5	5	5	5-6
	Yellow	5	5	5	5	5	5	5	6
	Red	5	5	5	5	5	5	5	5

B25	Blue	4-5	5	5	5	5	5	5	6
	Yellow	4-5	5	5	5	5	5	5	6
	Red	4-5	5	5	5	5	5	5	5-6

B30	Blue	5	5	5	5	5	5	5	5-6
	Yellow	5	5	5	5	5	5	5	6
	Red	5	5	5	5	5	5	5	5

**Table 7:** Tensile strength and tensile modulus of PP, PET and PP/PET blends.

Sample	Tensile strength (MPa)	Tensile modulus (MPa)
PP	31.40	1062.54
B12	33.05	1275.12
B15	34.16	1356.76
B20	33.84	1404.02
B25	30.08	1496.33
B30	29.15	1663.40
PET	61.67	2571.25
